# Putative Porcine Embryonic Stem Cell Lines Derived from Aggregated Four-Celled Cloned Embryos Produced by Oocyte Bisection Cloning

**DOI:** 10.1371/journal.pone.0118165

**Published:** 2015-02-13

**Authors:** Chawalit Siriboon, Yu-Hsuan Lin, Michel Kere, Chun-Da Chen, Lih-Ren Chen, Chien-Hong Chen, Ching-Fu Tu, Neng-Wen Lo, Jyh-Cherng Ju

**Affiliations:** 1 Graduate Institute of Basic Medical Science, China Medical University, Taichung, Taiwan, ROC; 2 Department of Animal Science, National Chung Hsing University, Taichung, Taiwan, ROC; 3 Division of Physiology, Livestock Research Institute, Council of Agriculture, Executive Yuan, Tainan, Taiwan, ROC; 4 Agriculture Technology Research Institute 1, Ln. 51, Dahu Rd., Xiangshan Dist., Hsinchu City, 300, Taiwan, ROC; 5 Department of Animal Science and Biotechnology, Tunghai University 181, Sec. 3, Taichung Harbor Road, Taichung, 407, Taiwan, ROC; 6 Core Laboratory for Stem Cell Research, Medical Research Department, China Medical University Hospital, Taichung, Taiwan, ROC; 7 Agricultural Biotechnology and Biotechnology Centers, National Chung Hsing University, Taichung, Taiwan, ROC; 8 Department of Biomedical Informatics, College of Computer Science, Asia University, Taichung, Taiwan, ROC; Baylor College of Medicine, UNITED STATES

## Abstract

We attempted to isolate ES cell lines using inner cell masses from high-quality cloned porcine blastocysts. After being seeded onto feeders, embryos had better (*P* < 0.05) attachment, outgrowth formation and primary colonization in both 2× and 3× aggregated cloned embryos (62.8, 42.6 and12.8% *vs*. 76.2, 55.2 and 26.2%, respectively) compared to the non-aggregated group (41.6, 23.4 and 3.9%). Effects of feeder types (STO *vs*. MEF) and serum sources (FBS *vs*. KSR) on extraction of cloned embryo-derived porcine ES cells were examined. More (17.1%) ntES cell lines over Passage 3 were generated in the MEF/KSR group. However, ntES cells cultured in KSR-supplemented medium had a low proliferation rate with defective morphology, and eventually underwent differentiation or apoptosis subsequently. Approximately 26.1, 22.7 and 35.7% of primary colonies were formed after plating embryos in DMEM, DMEM/F12 and α-MEM media, respectively. Survival rates of ntES cells cultured in α-MEM, DMEM and DMEM/F12 were 16.7, 4.3 and 6.8%, respectively (*P* > 0.05). We further examined the beneficial effect of TSA treatment of 3× aggregated cloned embryos on establishment of ntES cell lines. Primary colony numbers and survival rates of ntES cells beyond passage 3 were higher (*P* < 0.05) in those derived from TSA-treated 3× blastocysts (36.7 and 26.7%) than from the non-treated aggregated group (23.1 and 11.5%). These cells, remaining undifferentiated over 25 passages, had alkaline phosphatase activity and expressed ES specific markers Oct4, Nanog, Sox2, and Rex01. Moreover, these ntES cells successfully differentiated into embryoid bodies (EBs) that expressed specific genes of all three germ layers after being cultured in LIF-free medium. In conclusion, we have successfully derived putative porcine ntES cells with high efficiency from quality cloned embryos produced by embryo aggregation, and optimized the ES cell culture system suitable for establishing and maintaining ntES cell lines in undifferentiated state.

## Introduction

Embryonic stem (ES) cells, a pluripotent cell population with the capacity of self-renewal and differentiation into all body cell types and lineages, have great potential for use in regenerative medicine, research, and production of transgenic animals for xenotransplantation, e.g. the α-gal knockout pig [1–3]. Recently, ES or ES-like cells were derived from somatic cell nuclear transfer (SCNT) embryos in mice [4], rabbits [5], cattle [6], primates [7], and pigs [8,9]. The combination of SCNT and stem cell technology has numerous clinical applications in cell therapy and xenotransplantation, including mass-production of organs suitable for xenotransplantation [8].

Limited success of establishing porcine ntES cell lines is mainly attributed to the low efficiency of SCNT due to poor embryonic development, presumably as a result of incomplete cellular reprogramming and inadequate support from the *in vitro* culture system [10]. That the developmental potential of *in vitro*-derived blastocysts was lower than that of *in vivo* blastocysts [11,12], these cloned blastocysts had less total cell numbers and low ratio of inner cell mass (ICM) to trophectoderm (TE) cells than their *in vivo* counterparts [13]. Therefore, to improve cloning efficiency in pigs and to establish competent ntES cells, it is necessary to produce high-quality cloned blastocyst embryos. We previously reported that cloned porcine embryos treated with a histone deacetylation inhibitor (TSA) had enhanced histone acetylation and superior development compared to control embryos [14]. It is well known that reconstructed porcine embryos treated with TSA have an altered acetylation status of histone proteins, leading to enhanced reprogramming of the somatic genome and improved cloning efficiency [15,16].

The other crucial factor causing failure of embryo development is a suboptimal ratio of ICM and/or TE to total cell numbers [17,18]. However, in some studies, embryo aggregation improved embryo development *in vitro* [19]. Lee *et al*. [20] reported that aggregation of porcine embryos improved blastocyst rates, total cell numbers, and Oct4 expression. Similarly, we previously demonstrated that embryo aggregation improved developmental competency and the ratio of ICM to total cell numbers in cloned porcine embryos [29]. Although there were some claims that clone-clone aggregations did not improve embryo development in mice, it was noteworthy that total cell numbers and Oct4 expression levels were increased [21,22].

Putative porcine ES cells can be derived from early embryos, but they subsequently lose their pluripotency with repeated culture [23–27]. In that regard, most of these ES-like cells lacking definitive pluripotency markers failed to maintain their undifferentiated status [27], perhaps due to suboptimal culture conditions, since a great majority of the cells did not survive expansion during long-term culture. It is noteworthy that successful establishment of ES cells by SCNT with subsequent production of germline chimeras has apparently only been reported for mice and cattle [4,28].

The aim of this study was to improve the efficiency of establishing ntES cell lines by aggregation of genetically identical clones at the four-cell stage. After determining the beneficial effects of embryo aggregation and TSA treatment of cloned embryos on establishment of ntES cell lines, culture conditions were optimized to maintain putative porcine ntES cell lines and their pluripotency.

## Materials and Methods

### Animal Use and Ovary Collection

Oocytes were all aspirated from the ovaries collected from a local abattoir, where pigs were slaughtered with an electric shock and with guideline tightly stipulated by governmental standard operation procedure. This study was carried out in strict accordance with the Guideline recommended and approved (Permit Number: 97–95) by the Institutional Animal Care and Use Committee (IACUC) of the National Chung Hsing University.

### Production of Cloned and Parthenogenetic Embryos

Cumulus-oocyte complexes (COCs), aspirated from abattoir-derived ovarian follicles, were *in vitro* matured (IVM) in a 100-μL droplet of maturation medium (TCM 199 supplemented with 10% porcine follicular fluid and 10% FBS) containing gonadotropins (10 IU/mL hCG and 10 IU/mL PMSG) at 39°C under 5% CO_2_.

After IVM for 41 hours, matured oocytes with first polar body were incubated in 3.3 mg/mL pronase in HEPES-buffered TCM 199 supplemented with 33% fetal bovine serum (FBS) for 20 seconds and washed twice with HEPES-buffered TCM-199 (with 10% FBS; designated T10). After washing, oocytes were placed in 40 μL of T10 medium containing 2.5 mg/mL cytochalasin B (10 oocytes per droplet). For cloning with handmade cloning (HMC) or oocyte bisection technique (OBCT), oocytes were rotated with a fire-polished glass pipette to identify the membrane protrusion or first polar body for oriented bisection with a microblade, as described [29] under a stereomicroscope. After bisection, demi-ooplasts were washed twice in T10.

Cell fusion was performed with a two-step protocol consisting of two consecutive electric pulses. First, the enucleated cytoplast was transferred to the HEPES-TCM-199 droplet containing 1 mg/mL phytohaemagglutinin (PHA) for 5 seconds, and then moved to a T10 droplet holding fibroblasts. Each cytoplast was then allowed to pair with one fibroblast cell. The cytoplast-fibroblast pairs were incubated in the fusion medium (0.3 M mannitol and 0.01% PVA) for 20 seconds, and then transferred to the fusion chamber (two electrodes, 1 mm apart). Under a 0.6 kV/cm AC, cell pairs were aligned to the wire, with the fibroblasts farthest from the wire. Cell fusion was performed with one DC pulse at 2.0 kV/cm for 9 μseconds. The pairs were then transferred from the fusion chamber to the T10 drop and incubated for 1 hour before the second fusion.

For the second fusion, the remaining cytoplasts and the fused cytoplast-fibroblast pairs were transferred to the activation medium droplet (0.3 M mannitol, 0.1 mM MgSO_4_, 0.1 mM CaCl_2_ and 0.01% PVA) for equilibration. Then, they were aligned (0.6 kV/cm AC) with the fused pairs farthest from the wire, followed by a DC pulse (0.85 kV/cm) for 80 μseconds for the second fusion and initial activation. After elecrofusion and activation simultaneously, cytoplast-fibroblast triplets were incubated in T10 to allow complete fusion prior to chemical activation with 6-DMAP.

For production of parthenogenetic embryos, matured oocytes were activated by a DC pulse (2.2 kV/cm, 30 μseconds) in an activation chamber and then incubated in 6-DMAP for 4 hours under culture conditions as described [30].

### Embryo Aggregation

After parthenogenetic activation, reconstructed embryos were washed three times with 200 μL porcine zygote medium-3 (PZM-3). Parthenogenetic and OBCT embryos were cultured individually for 2 days up to the four-cell stage in the WOW system covered with mineral oil at 39°C in an incubator containing 5% CO_2_ in air. Embryo aggregation was performed with the four-celled embryos, and aggregated embryos were cultured continuously for an additional 5 days

### Preparation of STO and MEF Feeders

The STO fibroblasts were routinely cultured in DMEM supplemented with 10% FBS. Feeder cells were prepared using STO cells at less than 10 passages. After 80% confluency, the STO monolayers were exposed to 10 μg/mL mitomycin C (Sigma-Aldrich) for 2 hours, and then mitomycin C was washed off with PBS. Those STO cells were re-suspended in culture medium and seeded at a density of 0.8 × 10^5^ cells/cm^2^ in four-well dishes pre-coated with 0.1% gelatin.

Mouse embryonic fetal fibroblasts (MEFs) were isolated from fetuses at 13.5 day *post coitum*. The head, legs, and internal organs were removed by forceps and scissors in a 500 μL drop of 1% ABAM/PBS. After washing twice with 1% ABAM/PBS, the carcass was minced into small pieces with a surgical blade. Minced fetal tissues were washed by centrifugation (300 ×g for 10 min) and subsequently seeded into culture dishes with DMEM supplemented with 10% FBS at 37°C for 5–7 days. Derived MEF cells were cultured in DMEM medium supplemented with 10% FBS and sub-cultured when cells proliferated up to 80% confluence. At passages 3 and 4, once MEFs reached 80% confluence, they were treated with 10 μg/mL mitomycin-C (Sigma-Aldrich) for 3 hours to arrest mitosis, washed several times with PBS, and re-plated (0.8 × 10^5^ cells/well) in gelatin-coated, four-well culture dishes.

Inactivation of both STO and MEFs feeders was done 24 h before plating blastocysts or passaging pluripotent cell lines. Two hours before using the feeder cells, the medium was removed and replaced with pES cell medium.

### Outgrowth Culture Conditions

On Day 7, OBCT-derived and PA blastocysts were transferred from the culture dish to 200 μL drops of 3% ABAM/PBS for several washings. Thereafter, blastocysts were seeded onto a four-well dish containing a feeder layers with ES cell medium, according to experimental designs. The ES cell medium was supplemented with 1 mM L-glutamine (Sigma-Aldrich), 0.1 mM β-mercaptoethanol (Sigma-Aldrich), 10 mM MEM non-essential amino acids (Sigma-Aldrich), 0.03 mM adenosine (Sigma-Aldrich), 0.03 mM guanosine (Sigma-Aldrich), 0.03 mM cytidine (Sigma-Aldrich), 0.03 mM uridine (Sigma-Aldrich), 0.01 mM thymidine (Sigma-Aldrich), antibiotics (50 units/mL penicillin G, and 50 mg/mL streptomycin sulfate: Invitrogen), 5 ng/mL basic FGF, 20 ng/mL human recombinant LIF, 10 μM ROCK inhibitor (Y-27632; 1596–5; Biovision, Milpitas, CA, USA), and protein sources were either 15% FBS (Invitrogen) or 20% knock out serum replacement (KSR). Blastocysts were cultured for 5–7 days at 38.5°C in a 5% CO_2_ incubator and culture medium replaced with fresh ES cell medium every second day. When a colony enlarged to its full dimension, primary outgrowths were mechanically removed using a sterile glass pipette, and were transferred to a 100 μL drop of fresh medium for cutting into small pieces (30–50 cells per piece) with a microblade (ESE020, Bioniche Animal Health, Inc., Pullman, WA, USA) and mechanical pipetting under a stereomicroscope. Pieces of cells were then passaged and cultured on freshly prepared feeder-layers.

### Subculture, Freezing, and Thawing of ntES Cells

The ntES cell colonies were passaged every 8–10 days by picking up an individual undifferentiated ES cell colony and transferring it to 100 μL ES cell medium, where colonies were mechanically dissociated (microblade) into small pieces. The ES cell pieces were transferred and cultured onto new fresh feeders in pES cell medium. Medium was changed after 42 hours of culture and reattached ES cell colonies were examined.

Protocols for freezing and thawing of pES cells were as described [31]. Briefly, ES cell colonies were dissected into smaller pieces and suspended in 100 mL freezing medium, consisting of 10% dimethylsulfoxide (DMSO, Sigma-Aldrich) and 90% FBS, and then placed into a cryotube. The cryotubes were transferred to -20°C and kept for 4 hours, prior to preserving at -80°C overnight. On the following day, the cryotubes were directly plunged into -196°C liquid nitrogen for long-term storage. To thaw pES cells, cryotubes were removed from liquid nitrogen and submerged in a 37°C water bath for 30 seconds. The thawed cells were washed with 1 mL pES cell medium by centrifugation at 3,000 rpm for 3 minutes. Supernatant was removed and new fresh pES cell medium was added to re-suspend pES cells for plating on new feeders.

### Pluripotency Marker Assay

Alkaline phosphate (AP) staining was determined as described [32]. After culture, plates were rinsed twice in PBS and fixed in 4% formaldehyde in PBS for 15 minutes at room temperature; fixed cells were washed twice with PBS and then stained in AP solution for 15–30 minutes (until color development occurred). The AP staining buffer (100 mM Tris-HCl, 100 mM NaCl, and 50 mM MgCl_2_, pH 9.5) consisted of 0.25 M trizma maleate (Sigma T3128), 0.008 M MgCl_2_ (Sigma M8266), 0.17 g/L Fast Red TR salt (Sigma F8764), and 0.4 g/L α-naphthyl phosphate (Sigma N7255). Stained cells were observed under an inverted microscope after washing with PBS.

To analyze specific marker gene expressions, putative ES cell colonies were rinsed twice with Dulbecco’s phosphate buffered saline (DPBS, Gibco 21600–010) and then fixed in 4% paraformaldehyde for 30 minutes at room temperature. After fixation, colonies were rinsed three times with DPBS for 3 minutes each, and the cells were made permeable in 0.3% Triton X-100 (Sigma X100)/DPBS for 1 hour, followed by three washings with DPBS prior to incubation with blocking solution (DPBS + 2% skimmed milk) for 1 hour. Putative ES cells were incubated with primary antibodies, anti-Oct4 (Chemicon; AB3209), anti-Nanog (Abcam; ab115589), and anti-Sox2 (Abcam; ab97959). All antibodies were diluted in blocking solution (1:200) and incubated with samples overnight at 4°C after an additional three washes with 0.05% Tween-20/DPBS (15 minutes/wash). After incubation with primary antibody, ES cells were washed three times with 0.05% Tween-20/DPBS and then incubated with secondary antibodies for Oct4 (Alexa Fluor 546-conjugated goat anti-rabbit IgG; Invitrogen), Nanog (Alexa Fluor 594-conjugated donkey anti-goat IgG; Invitrogen), and Sox2 (Alexa Fluor 546-conjugated goat anti-rabbit IgG; Invitrogen) for 1 to 2 hours. Finally, fluorescent dye DAPI (1 μg/mL) was added to DPBS for nuclear staining, followed by two washes with DPBS for epifluorescence microscopy.

### Karyotyping

Chromosomes of established pES1 and pES3 cells were assessed at passages 20 and 25, respectively. The ES cells were incubated in medium supplemented with 0.5 μg/mL colchicine (Sigma Aldrich; C9754) overnight at 37°C in an incubator with 5% CO_2_ in air. After trypsinization and treatment with hypotonic KCl (0.56%) for 20 minutes, cells were placed in a hypotonic solution, fixed in a 3:1 (v/v) mixture of methanol and acetic acid, and spread on clean microscopic slides by gentle dropping. After staining with Giemsa (1:20 dilution; Sigma Aldrich) for 20 minutes, numbers of chromosomes were examined at ×1000 magnification.

### Gene Expression Analysis of ntES Cells

A small aliquot of the cell suspension was isolated and subjected to Reverse Transcription Polymerase Chain Reaction (RT-PCR) for expressions of genes listed in Table 1. Total RNAs were extracted using a total RNA extraction kit (Geneaid RT050). Cytoplasmic RNAs from pES cells were reverse-transcribed to generate first-strand cDNA. For this, 20 μL of total RNA, to which 2 μL of DNase I, 3 μL of DNase I 10× buffer, 0.25 μL of RNasin, 4.75 μL of ddH_2_O were added and reacted at 37°C for 25 minutes. After the first step, 1 μL EDTA and oligo-(dT) was added to the tube and reacted at 70°C for 5 minutes. Then, 1 μL RT enzyme (Promega M289A), 10 μL RT 5× buffer, 0.25 μL RNasin, 1 μL dNTP, 2.5 μL MgCl_2_, 3.25 μL ddH_2_O were added to the reaction and incubated for 1 hour. Aliquoted cDNAs were stored at -20°C until used.

**Table 1:** Primer Sequences and Related Information for PCR Analyses.

Target genes	Primers	Sequences (5’-3’)	Annealing temperature (°C)	Product size (bp)
Oct4	Forward	agtgagaggcaacctggaga	55	166
	Reverse	tcgttgcgaatagtcactgc		
Nanog	Forward	ttccttcctccatggatctg	56	214
	Reverse	atctgctggaggctgaggta		
Sox2	Forward	gccctgcagtacaactccat	61	216
	Reverse	gctgatcatgtcccgtaggt		
Rex01	Forward	cttcaaggagagcgcaaaac	52	299
	Reverse	tgtccccaatcaaaaatgct		
GATA4	Forward	ctcagaaggcagagagtgtg	59	281
	Reverse	ccgcattgcaagaggcctgg		
BMP-4	Forward	tgagcctttccagcaagttt	60	180
	Reverse	cttccccgtctcaggtatca		
β-III tubulin	Forward	ttccagctcacccactctct	52	214
	Reverse	tgtcgatgcagtaggtctcg		
β-actin	Forward	tgaaccctaaggccaaccgtg	60	267
	Reverse	tgtagccacgctcggtcagga		

For PCR, a total volume of 25 μL reaction solution containing 2 μL of the RT reaction mix were added along with 18.7 μL of ddH_2_O, 2.5 μL of 10× PCR buffer (2.5 mM MgCl_2_), 0.5 μL of dNTPs (10 mM each; Fermentas R0182), 0.3 μL of Taq DNA polymerase (5 U/μL, Geneaid), 0.5 μL forward primer (10 μM), and 0.5 μL reverse primer (10 μM). The PCR was done by specific primer sets derived from the GenBank database. Primer sequences including sense and antisense, annealing temperatures used in the PCR reactions, and the expected product size are listed (Table 1). An aliquot (4–10 μL) of PCR products were separated on a 1.5% agarose gel and visualized under UV light after ethidium bromide staining.

### Differentiation of pES Cells

For derivation of embryoid bodies (EBs), cells were cultured in pES cell medium without LIF and bFGF. The medium was refreshed daily for 10 days. Differentiation of EBs was confirmed, through RT-PCR screening for expression of markers (Table 1) associated with mesoderm (Bone Morphogenetic Protein-4, BMP-4), ectoderm (β-III tubulin) and endoderm (GATA4).

For spontaneous *in vitro* differentiation, day 7 EBs were mechanically dissociated and cells were plated directly onto gelatin-coated 4-well dishes in ES cell medium without inhibitors for adherent and spontaneous differentiation. After one week, cells were subjected to immunocytochemical analysis for embryonic germ layer lineages of the differentiated EB and were screened for the presence of differentiation markers: troponin I (for mesoderm; GeneTex; GTX113028), neurofilament light (NFL, for ectoderm; Millipore; AB9568), cytokeratin (for ectoderm; Sigma; C-2562), and α-fetoprotein (AFP, for endoderm; Santa Cruz; SC-8108).

### Experimental Designs

To determine optimal conditions for *in vitro* culture of porcine ICM cells, a series of experiments were conducted, as follows:

**Experiment 1: Competency of Aggregated Cloned and PA Porcine Blastocysts for Derivation of ES Cell Colonies.** Blastocysts were derived from aggregated OBCT embryos (1×, one-embryo; 2×, two-embryo aggregation; 3×, three-embryo aggregation) and 3× PA embryos at Day 7 of *in vitro* culture. After being washed four times with 3% ABAM in PBS, they were seeded onto mitomycin C-inactivated mouse STO feeder cells in a four-well culture dish containing ES cell medium (DMEM medium supplemented with 1 mM L-glutamine, 0.1 mM b-2-mercaptoethanol, 10 mM MEM nonessential amino acids, 0.03 mM adenosine, 0.03 mM guanosine, 0.03 mM cytidine, 0.03 mM uridine, 0.01 mM thymidine, 50 units/mL penicillin G, 50 mg/mL, 20 ng/mL bFGF, 20 ng/mL human recombinant LIF (hLIF), streptomycin, and 15% FBS). Blastocysts were cultured for 5–7 days to derive primary outgrowths and colonies.

**Experiment 2: Competency of Aggregated Cloned Blastocysts for Derivation of ES Cells Cultured with Various Feeder Cells and Sera.** Two feeder cells were used for culture of 3× cloned embryos to derive primary outgrowths and ES cell colonies. Thereafter, 3× cloned Day-7 embryos were either seeded onto mitomycin C-inactivated STO cells or MEF feeders in a four-well dish containing ES cell medium (DMEM medium supplemented with either 15% FBS or 20% KSR). Blastocysts were cultured for 5–7 days to derive primary outgrowths and colonies.

**Experiment 3: Competency of Aggregated Cloned Blastocysts to Derive ES Cell Lines in Various Culture Media.** Three culture media (DMEM, DMEM/F12 and alpha-MEM) were tested in this experiment. First, 3× cloned embryos of Day 7 were cultured onto mitomycin C-inactivated MEF feeder cells in a four-well culture dish containing DMEM, DMEM/F12 or α-MEM ES cell medium supplemented with 15% FBS. After 5–7 days of culture, numbers of primary outgrowths and ES cell colonies were observed and recorded.

**Experiment 4: Effect of TSA Treatment on Establishment of ntES Cell Lines.** In this experiment, 3× aggregated cloned blastocysts derived from TSA-treatment (for 24 hours) and non-treated embryos after activation were used to derive ntES cell lines, which were considered as being established if they survived repeated passaging and freezing. These ES cells were characterized by expressions of alkaline phosphatase activity, pluripotency marker genes (Oct4, Nanog, Sox2, and Rex01), and differentiation capacity.

### Statistical Analyses

All data were subjected to analysis of variance (ANOVA) using the General Linear Model (GLM) procedure in SAS, Version 9 (SAS Institute, Cary NC, USA), followed by Tukey’s test. Percentile data were arcsine-transformed before ANOVA and differences between treatment groups at *P* < 0.05 was considered significant.

## Results

### Experiment 1: Competency of Aggregated Cloned and PA Porcine Blastocysts for Derivation of ES Cell Colonies

**Fig 1:**
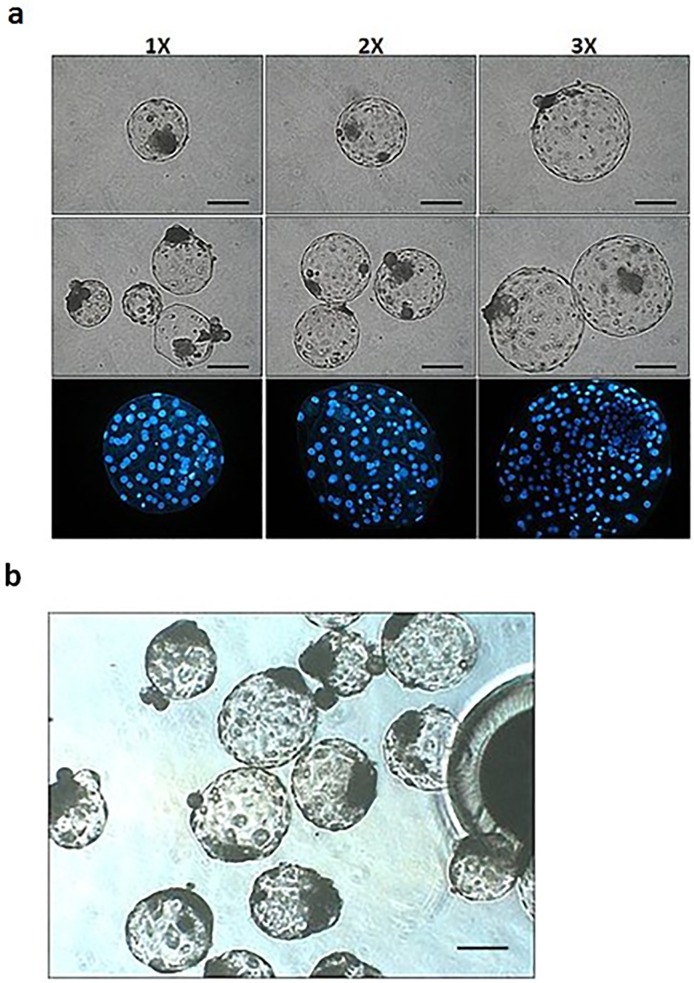
Morphologies of aggregated cloned and PA blastocyst embryos. (a) Day-7 cloned porcine blastocysts produced by OBCT and embryo aggregation. 1×: single cloned embryo; 2×: two cloned embryos were aggregated in a well, and 3×: three cloned embryos were aggregated in a well. (b) Day-7 3× aggregated porcine parthenogenetic embryos. Scale bar = 100 μm.

**Fig 2:**
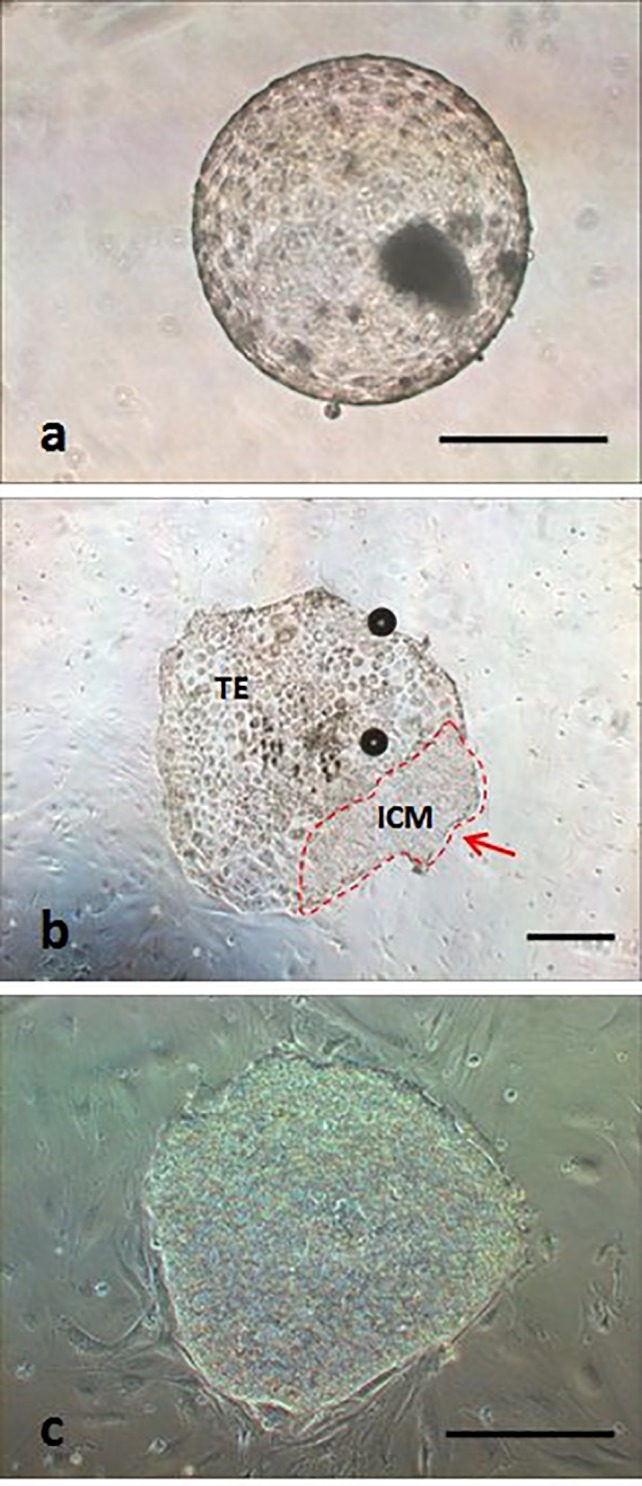
Formation of ntES cell colonies derived from the blastocyst embryos of the 3× cloned embryos. (a) A 3× blastocyst embryo (day 7) and (b) embryonal outgrowths with clearly ICM cells (red arrow) after culture for 5–8 days. (c) Typical morphology of a putative porcine ntES cell colony after passage. Scale bar = 100 μm.

Day-7 aggregated embryos derived by OBCT and PA processes (Fig. 1A-B) were tested for their ability to form outgrowths and primary ES cell colonies. Attached embryos in 2× and 3× aggregated groups (62.8 and 76.2%, respectively) performed better (*P* < 0.05) than those of 1× and 3×PA group (41.6 and 44.3%, respectively; Table 2). Outgrowths and primary colonies were detected 5–8 days after initial seeding. Similarly, more outgrowths were formed in 2× and 3× aggregated (42.6 and 55.2%, respectively) groups than those in 1× and 3×PA groups (23.4 and 25.3%; *P* < 0.05). In addition, 12.8 to 26.2% of primary colonies were observed in 2× and 3× groups, higher than those from 1× group (3.9%), but similar to that of 3×PA group (13.9%). The putative porcine ES cells had typical ES cell morphology, with compact colonies and distinct borders (Fig. 2B-C). However, most of the colonies only maintained typical ES morphology for one or two passages and then either differentiated or degenerated. However, five ES cell lines derived from the 3× group maintained proliferation without differentiation beyond three passages (Table 2).

**Table 2:** Competency of Aggregated Cloned and Parthenogenetic (PA) Porcine Blastocysts for Derivation of ES Cell Colonies.

Aggregated embryos	No. embryos			Primary colonies	No. lines > passage 3 (%)
	Total embryos	Attached embryos (%)	Outgrowth (%)		
1×	77	32 (41.6)^b^	18 (23.4)^b^	3 (3.9)^c^	0
2×	94	59 (62.8)^a^	40 (42.6)^a^	12 (12.8)^b^	0
3×	172	131 (76.2)^a^	95 (55.2)^a^	45 (26.2)^a^	5 (2.9)
3×PA	79	35 (44.3)^b^	20 (25.3)^b^	11 (13.9)^a^ ^b^	0

Number of replicates = 12

^a-c^Within a column, means without a common superscript differed (*P* < 0.05).

1× = one OBCT cloned embryo was cultured in a well; 2× = two OBCT cloned embryos were aggregated in a well; 3× = three OBCT cloned embryos were aggregated in a well; 3×PA = three PA embryos were aggregated in a well

### Experiment 2: Competency of Aggregated Cloned Blastocysts for Derivation of ES Cells Cultured with Various Feeder Cells and Sera

We further investigated the effects of various feeder cells and types of serum on deriving primary outgrowths and ES cell colonies. The Day-7 3× OBCT embryos were either seeded onto mitomycin C-inactivated mouse STO cells or MEF feeder cell layers in a four-well culture dish with ES cell medium (DMEM medium supplemented with either 15% FBS or 20% KSR). Various feeders and sera had no effect (*P* > 0.05) on ES cell establishment efficiency in terms of embryo attachment, outgrowths, and primary colony formation. More ES-like cell lines proliferated beyond passage 3 (17.1%) in MEF feeders and in ES cell medium supplemented with 20% KSR (Table 3). However, ES-like cell cultured in the medium supplemented with KSR had compromised pluripotency, defective morphology, and eventually became differentiated or subsequently died.

**Table 3:** Competency of Aggregated Cloned Blastocysts for Derivation of ES Cells Cultured on Different Feeder Cells with Sera.

Feeder cells	Serum*	No. embryos			Primary colonies	No. more than passage 3 (%)
		Total embryos	Attached embryos (%)	Outgrowth (%)		
STO	FBS	39	30 (76.9)	24 (61.1)	18 (46.2)	1 (2.6)
	KSR	37	22 (59.5)	15 (40.5)	11 (29.7)	3 (8.1)
MEF	FBS	36	28 (77.8)	21 (58.3)	17 (47.2)	2 (5.6)
	KSR	35	19 (54.3)	16 (45.7)	12 (34.3)	6 (17.1)

Number of replicates = 7

*ES cell medium was supplemented with 15% FBS or 20% KSR

### Experiment 3: Competency of Aggregated Cloned Blastocysts to Derive ES Cell Lines in Various Culture Media

Effects of various culture media on the growth of porcine ICM cells were compared (Table 4). There were no differences (*P* > 0.05) among treatment groups in embryo attachment rate, outgrowths, and primary colony formation. Percentages of ES-like cell lines beyond passage 3 in DMEM, DMEM/F12, and α-MEM medium were 4.3, 6.8, and 16.7%, respectively.

**Table 4:** Competency of Aggregated (3×) Cloned Porcine Blastocysts to Derive ES Cell Lines in Various Culture Media.

Medium	No. embryos			Primary colonies	No. more than passage 3 (%)
	Total embryos	Attached embryos (%)	Outgrowth (%)		
DMEM	46	25 (54.3)	18 (39.1)	12 (26.1)	2 (4.3)
DMEM/F12	44	24 (54.5)	17 (38.6)	10 (22.7)	3 (6.8)
α-MEM	42	26 (61.9)	18 (42.9)	15 (35.7)	7 (16.7)

Number of replicates = 7

### Experiment 4: Effect of TSA Treatment in 3× Aggregated Embryos on Derivation of Porcine ntES Cell Lines

The effect of TSA treatment in 3× aggregated embryos on the establishment of ntES-like cells is shown in Table 5. There was no difference in the efficiency with which outgrowth formation could be established from the TSA-treated blastocysts compared to the control (38.5 and 46.7%, respectively; *P* > 0.05). Primary outgrowths from the cloned embryos started to develop 4–6 days after plating onto MEF layers. Over the next 8–10 days, these colonies grew to 2–5 mm in diameter without changes in their morphology. The primary colony formation rate was greater (*P* < 0.05) in the TSA-treated group (36.7%) than the control (23.1%). Eight of the 11 (26.7% of plated embryos) primary colonies obtained from 30 TSA-treated 3× aggregated cloned embryos survived repeated passaging and freezing, and continued to grow without apparent changes in their morphology, thereby outperforming the control (11.5%, *P* < 0.05).

**Table 5:** Effect of TSA Treatment in 3× Aggregated Embryos on the Establishment of Putative Porcine ntES-Like Cell Lines.

TSA (30 nM)	Total embryos	Primary outgrowth (%)	Primary colonies (%)	No. survived beyond passage 3 (%)
-	26	10 (38.5)	6 (23.1)^b^	3 (11.5)^b^
+	30	14 (46.7)	11 (36.7)^a^	8 (26.7)^a^

Number of replicates = 4

^a,b^Within a column, means without a common superscript differed (*P* < 0.05).

**Fig 3:**
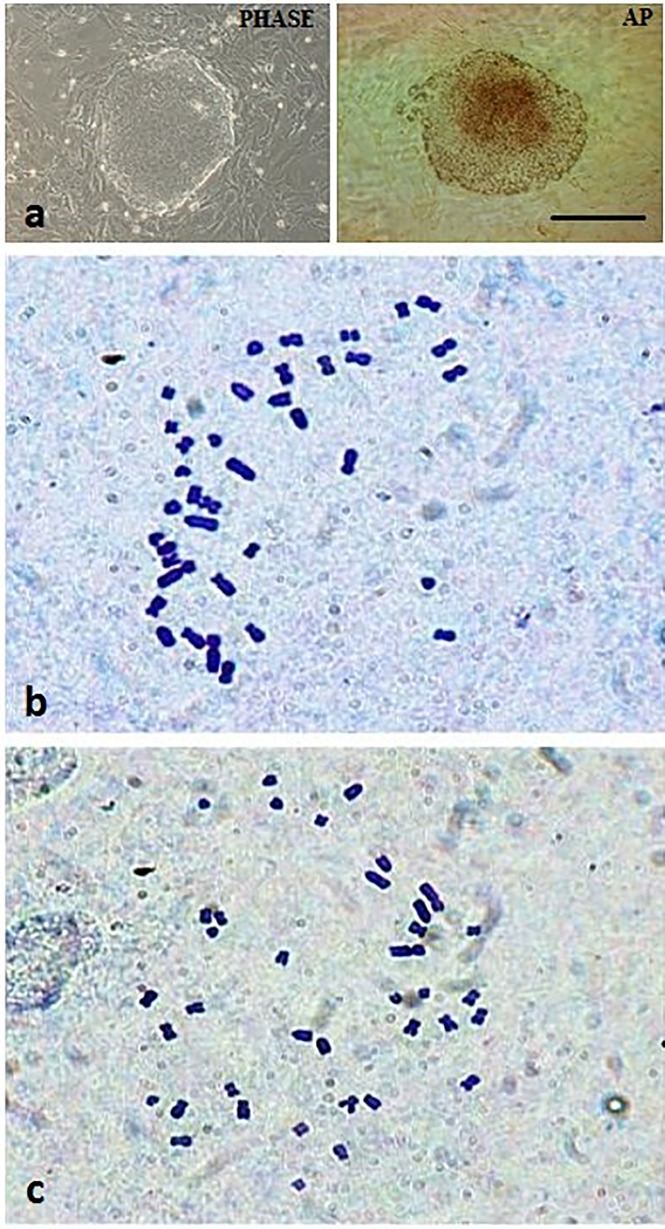
Alkaline phosphatase (AP) activity and karyotypes of ntES cells derived from aggregated cloned blastocysts. (a) A phase-contrast image of ntES cell colony with positive AP activity at passage 3. (b) Karyotyping by Giemsa staining of ntES cell line PES1 at passage 20 with 75% of normal karyotype, and (c) PES3 line at passage 28 with a normal karyotype ratio of 85%. Scale bar = 100 μm.

**Fig 4:**
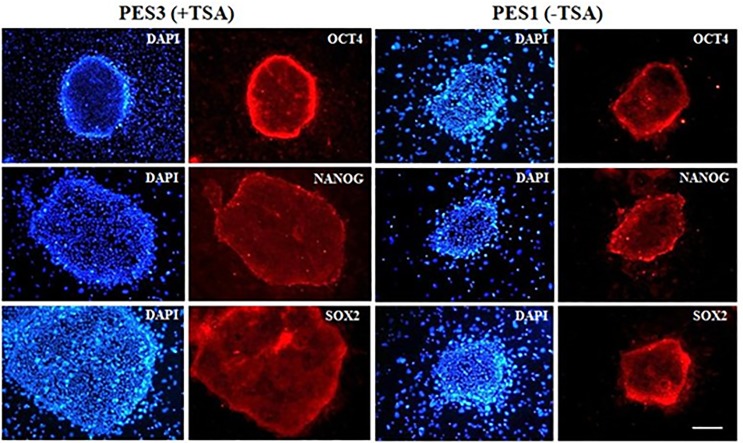
Immunofluorescence staining of pluripotency markers in ntES cell colonies derived from aggregated cloned embryos. Expressions of pluripotency markers (Oct4, Nanog and Sox2) are shown (red) in PES3 at passage 15 (left panel) and PES1 at passage 10 (right panel). Nuclei are stained with DAPI (blue). Scale bars = 100 μm.

**Fig 5:**
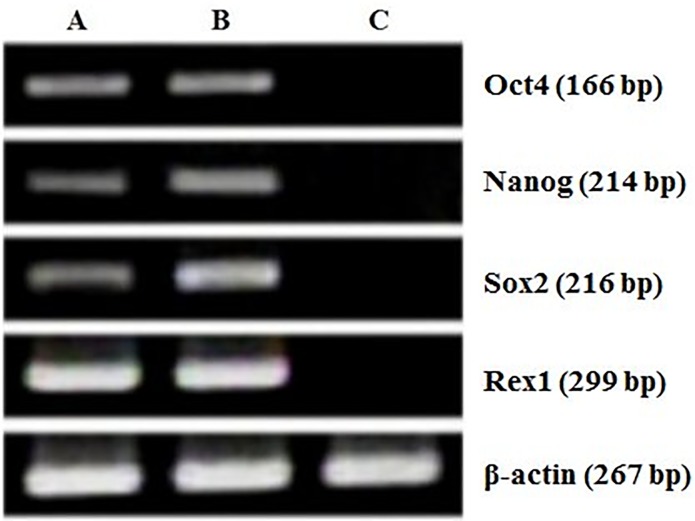
Characterization of ntES cells by real time RT-PCR analysis. Expressions of Oct4, Nanog, Sox2, and Rex01genes in (A) PES1 at passages 8, (B) PES3 at passages 15 and (C) pig fibroblast cells.

The ES-like cell colonies also had typical ES cell morphology including densely packed, small and round nuclei with well-defined boundaries and expression of AP activity (Fig. 3). Based on immunofluorescence staining and RT-PCR analysis, our ES-like cell colonies consisted entirely of those cells expressing Oct4, Nanog, Sox2, and Rex01 (Figs. 4 and 5).

**Fig 6:**
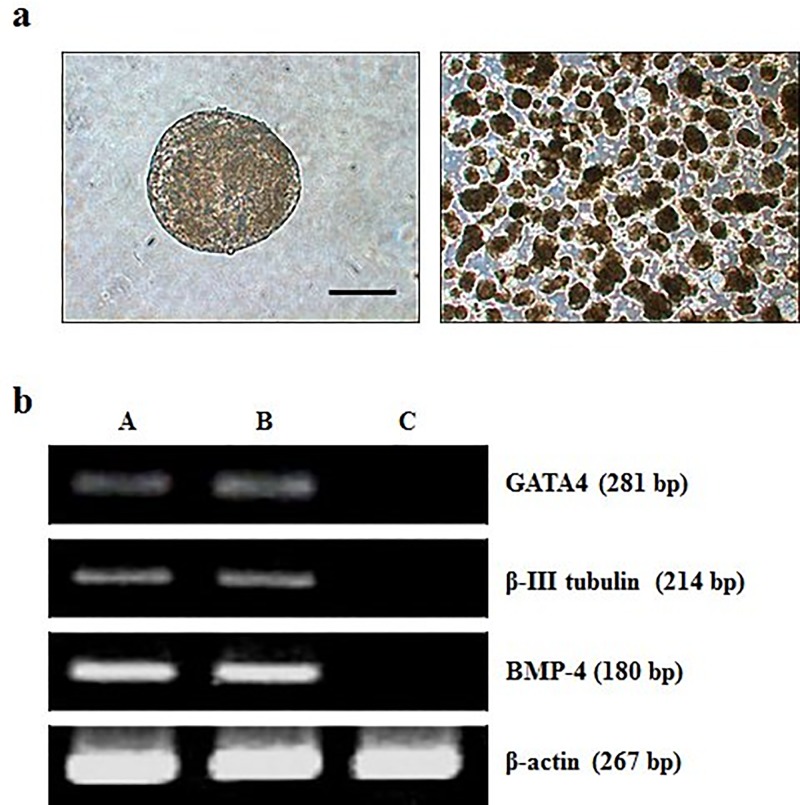
Differentiation of ntES cells in embryoid bodies (EB) and RT-PCR detection of genes typical for three germ layers. (a) Light microscopic images of EB cultured for 10 days. (b) Expressions of the genes representing all three germ layers, GATA4 (endoderm), β-III tubulin (ectoderm), and BMP-4 (mesoderm) in EB derived from PES1 (A), PES3 (B), and the negative control is represented by undifferentiated PES3 cells (C). Scale bars = 100 μm.

**Fig 7:**
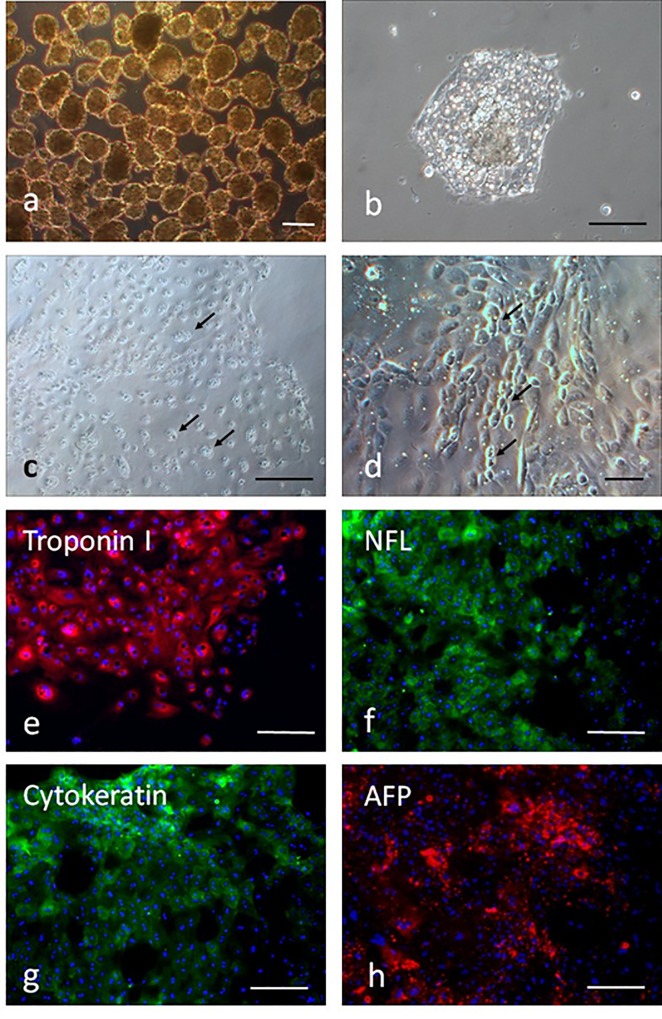
*In vitro* differentiation ability of porcine ntES cells after induction of EB formation. (a) Light microscopic images of EB cultured for 7 days. (b) EB attachment to gelatin-coated surface of culture dishes. (c) The neuron-like cells with obvious Nissl bodies after cultured for 3 days. (d) The epithelial-like cells morphology after cultured for 7 days. (e-h) Confirmation of the expression of differentiation marker troponin I (mesoderm), NFL and cytokeratin (ectoderm), and AFP (endoderm) from differentiated cells by the immunocytochemical analysis. Scale bars = 100 μm.

When the ES cells were cultured for 10 days on plastic petri dishes, cystic embryoid bodies formed (Fig. 6A). Expression levels of several ES cell specific genes of three embryonic germ layer cells were assessed for 10-day-old embryoid bodies. The mRNA transcripts of mesoderm (BMP-4), ectoderm (β-III tubulin) and endoderm (GATA4) were detected by RT-PCR (Fig. 6B). In addition, when day 7 EBs (Fig. 7A) were disaggregated and grown in gelatin-coated surface, they attached to culture dishes (Fig. 7B) after one day of culture. Some neuron-like cells showed obvious Nissl bodies (Fig. 7C) after cultured for 3 days, but became more epithelial cell-like morphology (Fig. 7D) after cultured for 7 days. Other cells showed the expression of troponin I, NFL, cytokeratin and AFP (Fig. 7E-H), respectively, indicating that subpopulations of cells representative of all three germ layers could be obtained.

## Discussion

The objective of the present study was to optimize production and culture conditions supporting primary culture of porcine ICM cells and stably passaged ES cell lines derived from cloned blastocysts. It is noteworthy that the success of establishing putative porcine ES cell lines is affected by several factors, including sources of embryos, ICM isolation methods, feeder cells, culture conditions, and culture media [33–39]. To establish putative porcine ES cell lines, blastocyst quality is among the most crucial factors affecting outgrowth and colony formation. Although porcine blastocysts have been produced with SCNT [14,15], in general, their quality was inferior to those produced *in vivo*. In this study, embryo aggregation technique provided the efficiency and quality to establishment of ntES cells, including embryo attachment rates, outgrowths, and primary colonies which explained that aggregation-derived blastocysts had enhanced cell-cell communication and thus facilitated development of outgrowths and also expression of pluripotency marker Oct4 of the established ntES cells [21]. Consistently, outgrowth formation was observed after culture of aggregated porcine-canine interspecies embryos onto feeder cells in ES cell medium [40]. Moreover, mouse parthenote (PA)-derived ES cells were established by aggregating PA embryos at the eight-cell stage [41].

The effects of feeders, sera, and media on putative porcine ES cell culture have been widely studied. It is generally agreed that ES cell lines established from different labs had diverse adaption to different culture conditions. In addition to providing options of culture systems from lab to lab, any subtle changes of potential factors affecting the outcome are worth to be re-examined and confirmed in a new system. Therefore, in this present study we attempted to derive and to optimize ES cell culture system by testing several crucial factors such as feeders, sera and media. The feeder cell is among the well-known essential factors for both establishment and maintenance of ES cell lines. One of the most important functions of feeders is to serve as an attachment matrix for cells and to secrete cytokines, e.g. LIF and other paracrine factors that may stimulate ES cell growth and inhibit differentiation [42]. In the present study, we demonstrated that the establishment of putative ES cell lines was more efficient on MEF than on STO feeders. This could be described that culture of putative porcine ES cells on STO feeders had a low attachment rate with less primary colony formation; furthermore, the majority of the cultured cells became differentiated after passaging [25,43]. According to Ropeter *et al*. [44], MEFs were better feeder cells for culturing ES cells which yielded more passages of putative ES cell lines. In addition, porcine embryonic fibroblast (PEF) cells and were also better than the STO feeders [42]. This phenomenon could be attributed to the reduced secreting activity of STO, since fewer microtubular structures were present in the STO cells [42]. Putative porcine ES cell lines have been derived by culturing cloned blastocysts onto MEF feeders for various passages [8,9]. Nevertheless, in some studies, STO feeder cells were also used to culture putative porcine ES cells derived from fertilized embryos for more than 10 passages [31,45].

It has been widely reported that FBS is beneficial to maintaining the morphology and pluripotency state of pig ES cells [31,38,46]. However, Goldsborough *et al*. (1998) also reported that FBS contained some major factors causing differentiation of ES cell lines. Recently, several studies have demonstrated that the defined serum-free supplement or knockout serum replacement (KSR) can be used to maintain ES cells in an undifferentiated state [8,9]. However, in the present study, there was no difference observed in the efficiency of deriving ES cell when embryos were plated in ES cell culture medium supplemented with either FBS or KSR. However, putative ES cells cultured in the medium supplemented with KSR showed a lower pluripotency marker expression, defective morphologies, and were readily differentiated during subsequent passages.

Although murine ES cells have been well-established, the culture system for putative porcine ES cells has apparently not been optimized [25,31,42,43,45,47–50]. Strojek *et al*. [51] reported that DMEM with 10% FCS yielded best results for ES cell establishment, although none of those cells were successfully subcultured for beyond five passages. Another study reported that two putative porcine ES cell lines were established using cloned porcine blastocysts cultured in DMEM/F12 supplemented with 20% KSR, which maintained the ES cells undifferentiated over 52 passages [8]. In the present study, various basal media (DMEM, DMEM/F12, and α-MEM) were compared using cloned and aggregated embryos produced by OBCT for ES cell derivation. We established ES cell lines in all three media tested, with α-MEM media having the greatest efficiency (16.7%, no. ES cell more than passage 3). In addition, we also used TSA in our cloning procedure to produce quality blastocysts for isolation of ES cell lines, as it enhanced blastocyst development of cloned embryos [14]. Previous work in mice and pigs also suggested that TSA could increase ES cell derivation rates in cloned embryos [9,52]. In the present study, higher proportions of ES cell lines were obtained from TSA-treated embryos (26.7%) compared to the untreated group (11.5%). This may be, at least in part, associated with the improved quality of cloned embryos, which was also supported by a recent study in human ntES cells (Tachibana et al., 2013). In their study, a human ntES cell line was derived from two high-quality oocytes donated by healthy volunteers [53].

In total, 11 cell lines were established in this study, of which the PES3 (derived from TSA-treated embryos) and PES1 (from control embryos) propagated rapidly with normal colony morphology upon repeated passaging. These cells expressed pluripotency marker genes (Oct4, Nanog, Sox2, and Rex01), and readily formed embryoid bodies in suspension culture, with the expression of genes representing all three germ layers (endoderm: GATA4; mesoderm: BMP-4; ectoderm: β-III tubulin). Moreover, the EB derived-differentiated cells exhibited various types of morphologies and expressed cell lineages of all three germ layers. Unfortunately, although teratoma formation is regarded as one of an essential pluripotency characteristics of ES cells, we failed to obtain teratomas after cell transfer into SCID mice (five replicates, 3–5×10^6^ morphologically undifferentiated ES cells), indicating these ES cell lines may not be sufficiently competent to become germline transmissible (data not shown). However, to date, there is no report of teratoma formation using pluripotent cell lines derived from either cloned or non-cloned porcine embryos. For instance, putative porcine ES cells generated by Anderson et al. (1996) failed to form teratomas via transplantation into the kidney capsule of athymic mice [54]. Recently, subcutaneously injection of porcine ES cells into non-obese diabetic/severe combined immunodeficient (NOD/SCID) mice were also unable to demonstrate teratoma formation [55]. Similarly, Kim *et al*. [8] reported that transplanted porcine ntES cells did not form teratomas in immuno-compromised mice, indicating more studies are required to derive truly pluripotent ntES cells in pigs, most likely by the use of small molecule signaling inhibitors or forced expression of pluripotency-related genes, such as Oct4, Sox2, Klf4, and c-Myc [56–59].

In conclusion, we have first successfully established and improved the efficacy of establishing putative porcine ES cell lines (pES1 and pES3 cells) using ICMs of quality blastocysts produced by OBCT and embryo aggregation. These cells can maintain undifferentiation and display typical ES cell pluripotency markers, EB forming capacity and differentiation into cell lineages of three germ layers. This report provides a new method to improve the efficiency of establishing ES cell lines which is of use in both agricultural and biomedical research. Nevertheless, it warrants more work to improve the quality of ES cell lines established, possibly by reversing these cells to their naïve status.
